# Highly automatic quantification of myocardial oedema in patients with acute myocardial infarction using bright blood T2-weighted CMR

**DOI:** 10.1186/1532-429X-15-28

**Published:** 2013-03-30

**Authors:** Hao Gao, Kushsairy Kadir, Alexander R Payne, John Soraghan, Colin Berry

**Affiliations:** 1 School of Mathematics and Statistics, University of Glasgow, Glasgow, G12 8QW, UK; 2 Centre for Excellence in Signal and Image Processing, Department of Electrical Engineering, University of Strathclyde, Glasgow, G1 1XW, UK; 3 BHF Glasgow Cardiovascular Research Centre, University of Glasgow, Glasgow, G12 8TA, UK; 4 Centre for Excellence in Signal and Image Processing, Department of Electrical Engineering, University of Strathclyde, Glasgow, G1 1XW, UK; 5 BHF Glasgow Cardiovascular Research Centre, University of Glasgow, Glasgow, G12 8TA, UK

**Keywords:** Myocardial oedema, Bright blood T2-weighted CMR, Rayleigh-Gaussian mixture model, Level set

## Abstract

**Background:**

T2-weighted cardiovascular magnetic resonance (CMR) is clinically-useful for imaging the ischemic area-at-risk and amount of salvageable myocardium in patients with acute myocardial infarction (MI). However, to date, quantification of oedema is user-defined and potentially subjective.

**Methods:**

We describe a highly automatic framework for quantifying myocardial oedema from bright blood T2-weighted CMR in patients with acute MI. Our approach retains user input (i.e. clinical judgment) to confirm the presence of oedema on an image which is then subjected to an automatic analysis. The new method was tested on 25 consecutive acute MI patients who had a CMR within 48 hours of hospital admission. Left ventricular wall boundaries were delineated automatically by variational level set methods followed by automatic detection of myocardial oedema by fitting a Rayleigh-Gaussian mixture statistical model. These data were compared with results from manual segmentation of the left ventricular wall and oedema, the current standard approach.

**Results:**

The mean perpendicular distances between automatically detected left ventricular boundaries and corresponding manual delineated boundaries were in the range of 1-2 mm. Dice similarity coefficients for agreement (0=no agreement, 1=perfect agreement) between manual delineation and automatic segmentation of the left ventricular wall boundaries and oedema regions were 0.86 and 0.74, respectively.

**Conclusion:**

Compared to standard manual approaches, the new highly automatic method for estimating myocardial oedema is accurate and straightforward. It has potential as a generic software tool for physicians to use in clinical practice.

## Background

The ischemic area-at-risk and myocardial salvage are determinants of prognosis in patients with recent myocardial infarction (MI) and potentially useful for risk stratification [1]. Myocardial oedema is due to water accumulation in injured myocardium [2], and T2-weighted cardiovascular magnetic resonance (CMR) depicts oedema which corresponds to the ischemic area-at-risk [3-6].

Assessment of myocardial oedema in a T2-weighted CMR scan requires judgment in order to interpret the presence and distribution of a hyperintense myocardial region in relation to the clinical history and other CMR findings. Beyond the initial clinical interpretation of the T2-weighted CMR scan, reliable quantification of oedema is challenging and time-consuming. The current manual approach for estimating myocardial oedema as a percentage of left ventricular (LV) mass initially involves delineation of the LV boundaries on each short axial scan from a stack of images extending from the base to the apex of the heart [7]. Subsequently, oedematous regions are identified and manually segmented within LV wall. This process typically involves adjustment of window and level settings and a threshold approach to delineate the hyperintense zone based on a difference in signal intensity of two or more standard deviations (SDs) from the mean signal intensity of an unaffected healthy region of interest [8]. All of these steps are potentially subject to observer errors. Estimation of the ischemic area-at-risk and myocardial salvage are relevant for clinical risk stratification, however area-at-risk and salvage are not usually measured in clinical practice because software tools are lacking. Therefore, computerized post-processing methods are needed for accurate oedema quantification.

Accurate LV boundary segmentation is the first essential step for computerized oedema quantification. A comprehensive review of these methods has been written by Petitjean et al. [9]. Although there are many studies on LV boundary segmentation from semi-automatic to fully automatic segmentation using cine CMR, very few studies have reported on segmentation of the LV wall with T2-weighted CMR. Stalidis et al. [10] used a contour deformable model to segment myocardial boundary from T2-weighted CMR. Their approach required manual selection of several seed points for initialization. Ciofolo et al. [11] proposed an automatic method to segment the myocardium boundary on late gadolinium enhancement (LGE) CMR with a multi-step approach by combining a geometrical template and shape prior.

Delineation of injured myocardium is the second essential step after LV segmentation. Even though LGE CMR and T2-weighted CMR both reveal myocardial injury (i.e. scar and oedema, respectively) with regional hyperenhancement, the segmentation challenges based on T2-weighted CMR are technically more demanding, particularly because of the comparatively reduced signal to noise levels associated with this method. LGE CMR has high diagnostic utility and is well established in clinical practice. Interpretation of LGE CMR by physicians has been facilitated by the development of computerized intensity thresholding techniques based on the standard deviation (SD) of healthy myocardial signal intensity [12-16]. However limited studies have been focused on automatic oedema quantification.

Kadir et al. [17] in our group used an automatic method described as a hybrid thresholding oedema sizing algorithm to automatically quantify oedema from T2-weighted CMR. The approach was based on simple intensity thresholding with the assumption that myocardial intensity had a Gaussian distribution, similar to the full width half maximum intensity algorithm [18]. Burchell et al. [19] suggested that the Otsu method could be used to replace the traditional SDs method based on 9 patients, and recently evaluated with LGE CMR by Vermes et al. [20]. Johnstone et al. [21] reported their results on oedema quantification based on dark blood T2-weighted CMR by fitting myocardium intensity histograms with a Gaussian mixture model. They showed that while the overall agreement between computerized and manual methods was good, there were discrepancies in area-at-risk estimation between the manual method and their method as measured in individual patients. Johnstone’s method was further developed by Sjogren et al. [22] by using a prior model of the maximal extent for the user defined culprit and based on the assumption that transmural ischemia occurs within the affected single coronary artery. By using regional analysis, their results showed improvements compared to Johnstone’s study with a mean oedema bias of -1.9±6.4% of LV volume compared to manual reference and higher degree agreement. Even so, the assumptions which underpin the model of Sjogren et al. [22] may have some limitations because of unmeasured physiological parameters, such as coronary collateral artery supply.

Until now, a robust and automatic approach involving LV boundary segmentation and delineation of area-at-risk has not been reported. Most studies have involved manual segmentation of LV boundaries [18,19,21,22], while Kadir’s method [17] was based on simple intensity thresholding. In this paper we propose a highly automatic scheme for quantification of myocardial oedema with short axis bright blood T2-weighted CMR images, and validated with standard manual reference, as used in clinical practice.

## Methods

**Figure 1:**
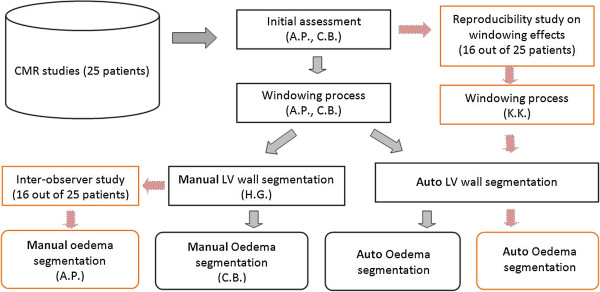
Study design for the proposed automatic oedema quantification scheme with observers involved in each step.

The strategy for the proposed oedema quantification scheme mainly includes CMR image acquisition and initial clinical interpretation, LV wall boundary segmentation, and oedema region delineation. Figure 1 schematically illustrates detailed steps from CMR image acquisition to final oedema quantification, with observers involved in each step.

### CMR image acquisition

CMR scans were performed on 25 consecutive patients (16 male, 9 female; mean (SD) age 55(12) years) within 48 hours of primary percutaneous coronary intervention (PPCI) for acute ST elevation myocardial infarction (STEMI). The study protocol was approved by the local ethics committee and all patients gave written informed consent. CMR was carried out on a Siemens MAGNETOM Avanto (Erlangen, Germany) 1.5-Tesla scanner with an 8-element phased array cardiac surface coil. The CMR protocol included breath-hold steady state free precession (SSFP) cine MRI and myocardial oedema imaging with bright blood turbo T2-weighted turbo spin echo steady state free precession (TSE-SSFP; ACUT2E) [23], and LGE phase sensitive inversion recovery (PSIR) MRI [24,25].

The ACUT2E acquisition involved the following parameters: acquisition time 7-12 s, matrix 192×192, flip angle 180°, echo time (TE) = 1.69 ms, bandwidth=789 Hz/pixel, slice thickness=6 mm with 4 mm gap, the voxel size was 1.9×1.9×6 mm^3^. Twenty nine coherent spin echoes (echo train length) were obtained per heartbeat and the time interval (echo spacing) between the 180° inversion pulses was 3.4 ms. The trigger pulse was 2 such that the data were acquired every second R-R interval. The ACUT2E method incorporated automated surface coil intensity correction by acquisition of a proton density gradient echo image interleaved with the T2-weighted acquisition every other heart beat. This image served as a proton density field map, which was used for surface coil intensity correction.

**Figure 2:**
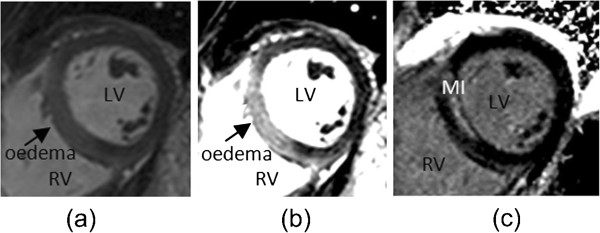
Example of short axis CMR images: (a) an initial bright blood T2-weighted oedema image and (b) transmural oedema more clearly revealed after user adjustment of window and level settings; (c) the corresponding LGE image reveals non-transmural hyperenhancement in the septum.

Myocardial infarction was imaged with a segmented PSIR turbo fast low-angle shot (PSIR-FLASH) [24,25] starting around 10 minutes after intravenous injection of 0.10 mmol/kg of gadoterate meglumine (Gd2+-DOTA, Dotarem, Guebert S.A.). Typical imaging parameters were: matrix=192×256, flip angle=25°, TE=3.36 ms, bandwidth=130 Hz/pixel, echo spacing =8.7 ms, trigger pulse=2, slice thickness=8 mm with 2 mm gap, the voxel size was 1.8×1.3×8 mm^3^. Figure 2(a) shows a typical image of bright blood T2-weighted myocardial oedema with the corresponding LGE image for MI in Figure 2(c).

### Clinical interpretation of T2-weighted CMR image

Two cardiologists (A.P. with 3 years CMR experience, C.B. with 5 years CMR experience) undertook the MRI analyses on a Siemens workstation with Syngo software. The MRI scans were obtained during usual patient care and the diagnostic reports were drafted by A.P., reviewed and verified by C.B. The window and level settings were also established at this time. The workstation allows user-defined selection of the level of the image display. Initial review of the ACUT2E scans involved standard adjustment of window and level signal intensity settings as previously described [7,8] (windowing process [26], Figure 2(b)) and routinely performed in our laboratory. Myocardial tissue with signal intensity at least ×2 SD above the mean signal obtained in the remote non-infarcted myocardium was considered to have evidence of oedema.

For the purpose of this analysis, all images were coded and de-identified. Short axis images from base to apex of the LV were selected for each patient with two exceptions: (1) slices from the base were excluded if the circumference of the heart included the mitral valve orifice or LV outflow tract; (2) the most apical slices were excluded if the endocardial border of the LV cavity was unclear. According to these criteria, of 221 images, 171 short axis T2-weighted MR images from 25 patients were suitable for analysis (approximately 7 slices per patient), of which 142 slices with visual evidence of myocardial oedema, and 11**7** out of 142 T2-weighted CMR scans were obtained from slice positions which also had evidence of infarction revealed by LGE.

### T2-weighted CMR image analysis

All analyses were performed using in-house Matlab programs. The signal intensity of all MR slices was re-scaled and standardized in the range 0–255 as done by Syngo software in the Siemens workstation according to Eq. 1.

(1) $$I=\left\{\begin{array}{l} 0\;I\;<\;I_{c}\;-I_{w}/2\\ 255*\frac{I-\left(I_{c}-I_{w}/2\right)}{I_{w}}\;I_{c}-I_{w}/2\;\underset{\leq }{<}\;I\;\underset{\leq }{<}\;I_{c}+I_{w}/2\\ 255\;I\;>\;I_{c}-I_{w}/2 \end{array}\right)$$

where *I* stands for the original pixel intensity, *I*_*c*_ and *I*_*w*_ are the centre and width of the intensity window, which are available from the DICOM header.

### Manual quantification of oedema

Manual contouring of the LV wall and oedema was carried out by a Matlab program to provide the reference dataset. Firstly, LV wall boundaries on bright blood T2-weighted CMR were manual delineated by H.G. (3 years in CMR image processing) on 171 slices in total; secondly, C.B. performed the manual oedema quantification for all the 25 patients with super-imposed LV wall boundaries from H.G., which was the reference data for the proposed automatic approach. Separately, A.P. did the manual oedema quantification for 16 patients and the results of this analysis were used for the inter-observer study. LGE and cine MRI scans were available if necessary to aid the segmentation of oedema by providing improved anatomical and functional information and care was taken to exclude blood pool signal adjacent to the sub-endocardium. For each patient, voxels in oedematous regions from base to apex were counted for oedema mass, and then oedema extent in each patient was defined as the percentage of the whole LV mass calculated from the T2-weighted slices from base to apex. The manual oedema delineation method by C.B. and A.P. has been validated and routinely performed in the clinical settings [7,8,27].

### Automatic quantification of myocardial oedema

The same short axis bright blood T2-weighted MR images were used for the proposed automatic approach. There were two main stages. Stage 1: automatic LV wall segmentation on all short axis images; Stage 2: automatic oedema quantification with the inputs from stage 1 on the slices with oedema presence. Since the windowing process was involved before exporting these DICOM images (the human inputs), while the other steps were fully automatic, therefore the proposed approach was considered to be highly automatic.

#### Stage 1: Automatic LV wall segmentation

The LV endocardial and epicardial wall boundaries were defined for each slice by using an automatic approach based on a variational level set method. A detailed theoretical description of the approach is provided in Additional file 1, refer to [28] for more details. In brief, the procedure involves:

i) automatic LV centre point detection in each slice [29] and

ii) variational level set method without re-initialization [30] for LV wall segmentation by using the LV cavity centre information from the previous step. Papillary muscles are excluded from LV wall.

#### Stage 2: Automatic segmentation of myocardial oedema

The automatic method for myocardial oedema delineation consists of four main steps:

i) Rayleigh-Gaussian mixture model fitting for the intensity histogram of LV wall;

ii) Thresholding operation;

iii) Morphological filtering;

iv) Oedema region feature analysis.

**Figure 3:**
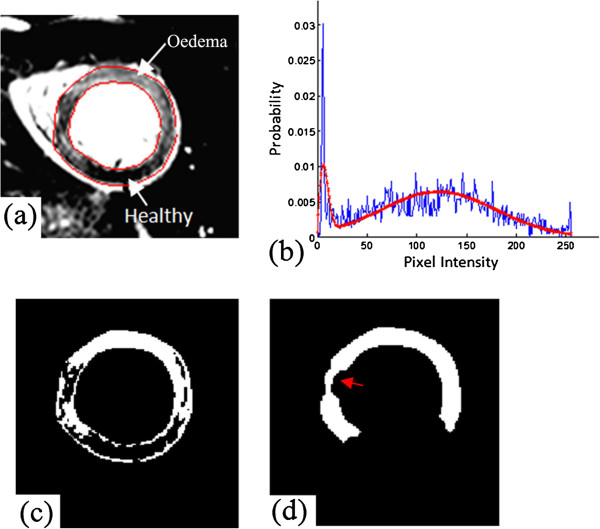
Automatic oedema delineation: (a) an Oedema image with LV boundaries delineated by the automatic approach; (b) myocardium intensity distribution (blue) and fitted by Rayleigh-Gaussian mixture model (red); (c) binary image after thresholding process (white regions are potentially oedema); (d) oedema regions (white) after morphological filtering.

**Step 1: Rayleigh-Gaussian mixture model fitting:** The widely used simple xSD thresholding, methods used by Kadir [28] and Johnstone [21] are based on the assumption that myocardial intensity has a Gaussian distribution for healthy and oedema regions. While in the presence of noise, the magnitude MR image intensity is governed by a Rician distribution [31]. Image intensity can be approximated by a Rayleigh distribution when intensity values are close to zero, and tends to be a Gaussian distribution when values are high. In a short axis image obtained with T2-weighted CMR (shown in Figure 3(a)), unaffected myocardium appears dark while pathological tissue appears bright. Therefore it is possible to represent the healthy and pathological myocardium by using Rayleigh and Gaussian distributions respectively. In order to fit a Rayleigh-Gaussian mixture to the intensity histogram of LV wall, the intensity distribution function *f*_*myoc*_ of myocardium is defined as follows [16]:

(2) $$f_{\mathrm{myoc}}\left(I\right)=a_{n}\cdot f_{n}\left(I+\alpha_{\mathrm{shift}},\sigma_{n}\right)+a_{e}\cdot f_{e}\left(I,\mu_{e},\sigma_{e}\right)$$

(3) $$f_{n}\left(I,\sigma_{n}\right)=\frac{I}{\sigma_{n}^{2}}e^{-\frac{I^{2}}{2\sigma_{n}^{2}}},I\;\underline{>}\;0$$

(4) $$f_{e}\left(I,\mu_{e},\sigma_{e}\right)=\frac{1}{\sqrt{2\pi \sigma_{e}^{2}}}e^{-\frac{{\left(I-\mu_{e}\right)}^{2}}{2\sigma_{e}^{2}}}$$

Eq. 2 is the Rayleigh-Gaussian mixture model, Eq. 3 and Eq. 4 represent the Rayleigh and Gaussian distributions respectively. In Eq.1 *a*_*n*_ and *a*_*e*_ are the weighting parameters for the two models; *σ*_*n*_ is the Rayleigh distribution parameter; *μ*_*e*_ and *σ*_*e*_ are the mean and standard deviation of the Gaussian distribution; *I* stands for myocardium intensity in LV wall. *α*_*shift*_ represents the intensity shift during MR image windowing and intensity adjustments. In a similar manner to the signal processing during LGE CMR acquisition used by Elagouni et al’s study [16], this shift occurs because the windowing procedure makes the unaffected healthy myocardium as dark as possible for maximizing contrast and the identification of bright oedematous myocardium. The estimation of the distribution parameters is achieved by maximizing the likelihood according to myocardium intensity histogram with an Expectation Maximization algorithm [32]. Figure 3(b) shows the corresponding fitting results for the oedema image in Figure 3(a), which has been well represented by Eq. 2

**Step 2: Thresholding operation:** a threshold value was defined to classify healthy and pathological tissues after the fitting process. The mean of the Rayleigh distribution is

(5) $$\mu_{n}=\sigma_{n}\sqrt{\frac{\pi }{2}}$$

A fuzzy membership map *I*_*map*_ was defined according to *μ*_*n*_ and *μ*_*e*_ as,

(6) $$I_{\mathrm{map}}=\left\{\begin{array}{l} 0,\;I\;\underset{\leq }{<}\;\mu_{n}\\ \frac{I-\mu_{n}}{\mu_{e}-\mu_{n}},\mu_{n}\;<\;I\;<\;\mu_{e}\\ 1,\;I\;\underset{\leq }{>}\;\mu_{e} \end{array}\right)$$

For each pixel, if intensity value *I* is less than *μ*_*n*_, then the pixel is classified as unaffected myocardium, and *I*_*map*_=0; if *I* is greater than *μ*_*e*_, then it is classified as oedema tissue, and *I*_*map*_=1; *I*_*map*_ varies linearly from *μ*_*n*_ to *μ*_*e*_. In this study, a membership value of *I*_*map*_ ≥ 0.7 was defined as the thresholding value for the regions of oedema. The value of 0.7 was chosen based on a pilot study of 4 patients by maximizing the agreement with manual delineations. Furthermore a parameter study by varying the thresholding value from 0.6 to 0.9 is summarized in the result section, which confirms 0.7 is optimal for this study.

**Step 3: Morphological Filtering:** After thresholding, segmented regions of myocardial oedema were processed using an alternative sequential morphological filtering which is a robust approach to preserve topology [33]. Sequential morphological filtering includes:

i) a morphological closing operation with a small kernel (disk shape with size of 2 pixels) for removing the noise and false positives;

ii) an opening process with the same kernel as in the first step;

iii) a closing process with a bigger kernel (disk shape with size of 5 pixels) is applied to connect isolated regions if close enough.

Since a region of myocardial oedema may also include dark zones of reduced signal, such as arising from myocardial haemorrhage [27], the closing operation helps fill the dark regions inside oedema region. Figure 3(c, d) show an example of oedema results extracted using our proposed method with thresholding followed by the morphological filtering respectively. Figure 3(d) is seen to be much smoother with better defined regions compared to Figure 3(c). However for the large dark regions inside oedema, the closing process may not be able to fill them, indicated by an arrow in Figure 3(d).

**Figure 4:**
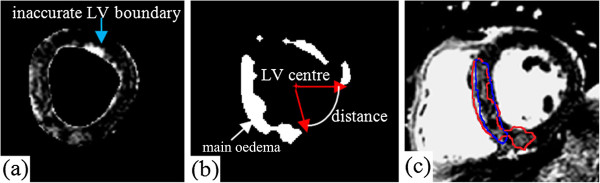
Automatic oedema delineation with feature analysis: (a) an oedema image with LV boundaries; (b) potential oedema regions after morphological filtering; (c) final oedema regions after region feature analysis (enclosed by red line), close to the manual delineation (blue line) from C.B.

**Step 4: Oedema region feature analysis:** Given the regional location of myocardial infarction following coronary artery occlusion, a short axis slice typically has one area of oedema (Figure 3(d)). However due to imaging intensity variations, small bright regions may occur in healthy myocardium without oedema. Bright areas such as these or others due to inaccurate LV wall boundary segmentation are apparent in Figure 4(b). The segmented regions in Figure 4(b) are labelled using 8-neighbor connectivity analysis and the largest hyperintense region is considered to be the main area of injury. As in Figure 4(b), except for the main oedema region, three other regions in the side opposite to the main oedema region are identified. Therefore additional analysis is required to check whether these regions should be considered to be oedema according to the relative size and the arc distance to the main oedema region. The arc distance from one of these regions to the main oedema region is defined in degrees according to the LV cavity centre, as shown in Figure 4(b). In line with the approaches used for segmentation in previous studies [15,21], our region feature analysis procedure is:

i) The region is considered to be oedema if the area of the region is greater than two fifths of the main oedema region;

ii) The region is considered to be oedema if the area of the region lies in the range from one fifth to two fifths of the main oedema region, and the arc distance from the region to the main oedema region is less than 20 degrees;

iii) The region is taken to be oedema if the area of the region is less than one fifth of the main oedema region, and the arc distance from the region to the main oedema region is less than 10 degrees.

Figure 4(c) shows an example of the final result of the oedema region (enclosed by the red line) after the area feature analysis based on the oedema image in Figure 4(a), superimposed with manual segmentation from C.B. (enclosed by the blue line). After feature analysis, the detected oedema is much closer to the manual delineation than without feature analysis, though discrepancy exists.

### Reproducibility study

In order to study inter-operator variations in manual oedema quantification, 16 out of 25 patients were manual segmented again by A.P., and compared with manual results from C.B. Furthermore, because the manual windowing of oedema images after CMR acquisition could cause variations on the final oedema results, therefore the same 16 patients were re-evaluated by K.K. (3 years’ experience in oedema image analysis) blinded to the previous dataset for the variation assessment on the effects resulting from the windowing process, followed by automatic LV boundary segmentation and oedema delineation, finally the newly obtained oedema results were compared with the results from the windowing process done by A.P.

### Statistical analysis

The segmented regions were categorized as either oedematous or healthy. The spatial overlap between the results from the proposed approach and manual segmentations was assessed using the Dice similarity coefficient (Dsc), defined as

(7) $$\mathrm{Dsc}\left(A,B\right)=\frac{2\left(A\cap B\right)}{A+B}$$

where ∩ represents the intersection of the two regions and *A+B* represents the sum of the areas/volumes. Dsc(A,B)=1 indicates a perfect overlap (agreement) between A and B and Dsc(A,B)=0 means no overlap between A and B. Zijdenbos et al. [34] suggests that a Dice similarity coefficient >0.7 indicates good agreement, therefore in our study, 0.7 was considered a criterion for reasonably good agreement. The mean perpendicular distance between paired automatically segmented and manual boundaries [9] and Dice similarity coefficient were compared for assessing LV boundary segmentation accuracy.

The agreement for oedema quantification was done by comparing oedema extent (counting oedema from base to apex in each patient) with manual oedema segmentations. Oedema extent was defined as% of LV mass for each patient, in which the LV mass was calculated by reconstructing 3D LV wall geometry from corresponding boundaries on those T2-weighted images, with a density of 1.05 g/cm^3^. Furthermore, quantification of oedema by Kadir’s method [17] was implemented to compare with our approach on same slices. The same morphological filtering and regional feature analysis were applied to make fair comparison. All statistical analyses were performed using Matlab and an alpha of 0.05 was taken as significant.

## Results

### left ventricular wall segmentation

**Figure 5:**
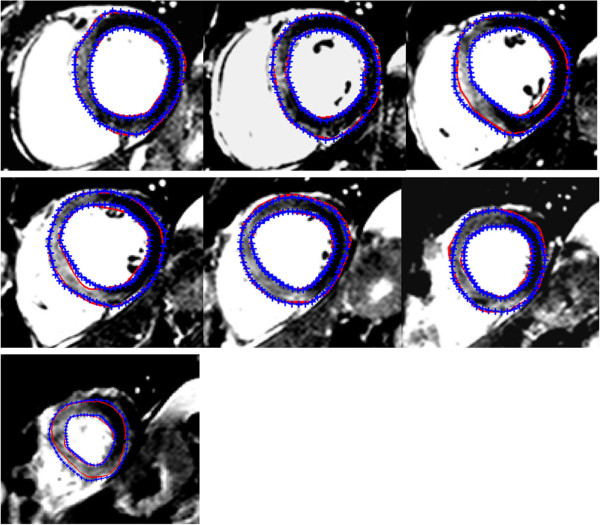
Example of left ventricular boundary segmenation by the automatic approach from base to apex (blue: manual; red: automatic).

**Figure 6:**
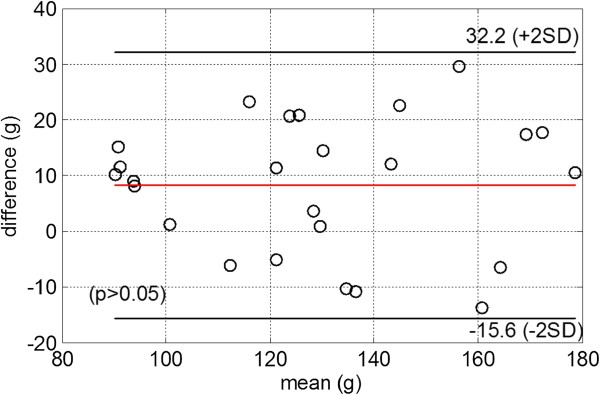
Bland-Altman plot of LV mass between manual results and the automatic approach, the difference is defined by auto – manual.

Figure 5 shows an example of automatic LV wall boundary segmentation on 7 short axial images from base to apex from one patient superimposed with manual segmentation. Qualitatively, the LV wall delineated by our approach appears close to the manual segmentation. The mean perpendicular distance for the 171 slices between the automatic approach and the manual segmented LV boundaries is 1.05±0.4 mm for the endocardial boundary and 1.56±0.68 mm for the epicardial boundary. The distance is larger on the epicardial wall than on the endocardial wall due to the poorer contrast between epicardium and surrounding tissue. The mean perpendicular distance from our approach is comparable to those from other studies on LV wall segmentation accuracy, which is in the range 1-2 mm [9]. The average Dice similarity coefficient of the LV wall region is 0.86±0.05 for the 171 slices. For each patient, the averaged Dice similarity coefficient of the 3D LV wall geometry is 0.87±0.02, the average LV mass is 125±28 g for manual method and 133±28 g for the automatic approach. Figure 6 is the Bland-Altman analysis between the manual and automatic results with a mean bias of 8±12 g (the automatic approach - manual). The comparison suggests that good accuracy for LV wall segmentation can be achieved with our automatic approach.

### Results of oedema quantification

**Figure 7:**
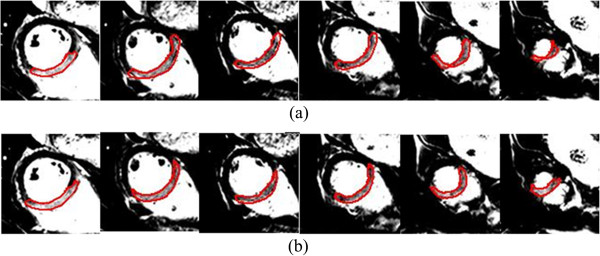
Example of oedema segmentation from (a) the proposed automatic approach and (b) manual method: C.B.

Since the actual amount of oedematous myocardium for each patient was not available, a standard validated approach for estimating the extent of myocardial oedema was adopted [7,8,27] for the comparison with the automatic approach. A parameter study of the thresholding operation for automatic oedema delineation was conducted first by varying the thresholding value of *I*_*map*_ from 0.6 to 0.9 (Eq. 5), the results are summarized in Table 1. The thresholding value of 0.7 gives better results than others in general, and when the threshold value increases, the oedema extent decreases and vice versa. Figure 7(a) shows oedema delineated using our approach with manual delineation from C.B. (Figure 7(b)) for one patient from basal region to apex, 6 slices in total. Based on visual inspection from Figure 7, there is good agreement between the automatic approach and the manual segmentation approach for definition of the oedema region.

**Table 1:** Parameter study on the thresholding value (25 patients)

**Thresholding value**	**Oedema extent**	**Difference related to C.B.**	**Dice similarity coefficient (C.B.)**
0.6	31±9%	6±5%	0.73±0.06
0.7	28±8%	3±3%	0.74±0.06
0.8	25±7%	4±4%	0.73±0.07
0.9	21±6%	6±5%	0.7±0.08

C.B.: cardiologist.

**Figure 8:**
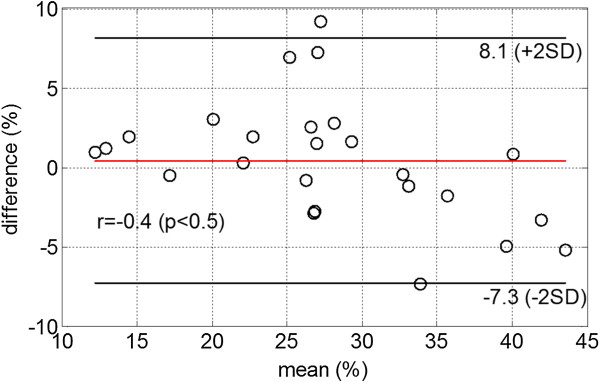
Bland-Altman plot of oedema extent between manual results and the automatic approach, the difference is defined as auto – manual.

Hyperintense oedematous regions of interest were successfully quantified in 142 oedema MR images. The mean oedema extent from the automatic approach is 28±8%, close to the results from manual quantification (C.B. 27±10% (*p*=0.07), with an absolute difference of 3±3%. The mean Dice similarity coefficient based on oedema volume overlap is 0.74±0.06. Figure 8 is the Bland-Altman analysis between the manual and our automatic results. The mean bias for oedema extent related to the whole LV mass is 0.4±4% (the automatic approach - manual), with a weak correlation to the mean oedema mass extent (*r=-0.4, p=0.03*), indicating the automatic approach tends to overestimate oedema region when reference oedema region is small, and underestimate vice versa.

The results of the comparison of the volumetric extent of oedema by manual segmentation (C.B.) and the automatic approach using Kadir’s method [17] are summarized in Table 2. Kadir’s method underestimates the oedema extent (*p=0.02*) with Dice similarity coefficient of 0.7; while the new automatic approach generates the closest results compared to the manual oedema delineation.

**Table 2:** Oedema comparisons for different methods (25 patients)

**Oedema mass extent**			
	C.B.	Auto	Kadir’s method
mean	27±10%	28±8%	22±7%
**Dice similarity coefficient based on volume overlap (related to C.B.)**			
	C.B.	Auto	Kadir’s method
mean	~	0.74±0.06	0.7±0.09

C.B.: cardiologist; Auto: the automatic approach.

### Reproducibility

In the inter-observer study of manual oedema quantification with same computed LV wall boundaries, the averaged difference in the volumetric extent of oedema (% of LV) between A.P. and C.B. is 3±2%, and the mean Dice similarity coefficient between A.P. and C.B. is 0.85±0.03. Table 3 shows the windowing effects on oedema quantification. The extent of oedema is similar between the two processes (*p=0.9*) with an averaged Dice similarity coefficient of 0.84±0.07, close to the average value between A.P. and B.C. (0.85±0.03 from the inter-observer study). The difference in the extent of oedema from the two independent windowing processes is 3±3%, which is close to the difference between A.P. and C.B. for the 16 patients (3±2%) from the inter-observer study.

**Table 3:** Effect from the windowing process (16 patients)

	**Oedema mass extent**		**Dice similarity**
	Auto	Auto^1^	Auto vs. Auto^1^
Mean	29±7%	29±10%	0.84±0.07

Auto: windowing process by A.P.

Auto^1^: windowing process by K.K. 6 months later.

The averaged processing time for one patient is 40±6 seconds on a laptop (2.40 GHz Inter Core i5 CPU, 4GB memory), compared to the processing time for manual oedema quantification (range 360 to 420 seconds for an experienced cardiologist). In the proposed approach, the total average times for LV segmentation and oedema delineation are 34±6 and 6±0.9 seconds for each patient, less than one minute overall.

## Discussion

We have developed and tested a highly automatic method for estimating the extent of myocardial oedema by using bright blood T2-weighted CMR in patients with acute myocardial infarction. Physician judgment is retained as the initial step in image analysis and following this initial evaluation, images are subjected to automatic segmentation of the LV wall and oedema territory. High levels of agreement for the proposed approach are found when compared to a standardized and validated manual approach for myocardial oedema analysis on T2-weighted CMR (Dice similarity coefficient >0.7). Furthermore our new approach is also seen to outperform two other computerized methods [17,21]. Although the results from Sjogren et al. [22] have a higher Dice similarity coefficient than our approach, their results were obtained from manually segmented LV boundaries. Recognising the value of clinical judgement, we retained user input as a key first step in the process. Thus, observer judgement is initially required to assess for the presence and distribution of hyperintense myocardial regions and place these observations in a clinical context (e.g. acute myocardial infarction). Thus clinical judgement is the first step in image interpretation prior to automatic analyses, which is why the proposed method is considered as highly automatic rather than fully automatic.

LV wall boundary segmentation (semi-/fully automatic) with CMR has been extensively studied [35-37]. However accurate LV segmentation remains a challenge especially for quantitative analysis of global and regional cardiac function. In the current study, a level set method without re-initialization was used for LV wall segmentation. The comparison with manual segmentation shows that this method can generate good LV wall boundaries for further oedema quantification. Unlike previous studies [16,21,22], in which the computerized method for oedema delineation required manual defined LV wall boundaries (time consuming, possible large inter/intra-observer variations), our approach directly links the oedema delineation to the automatic LV wall segmentation procedure. However the uncertainties arising from automatic LV wall segmentation will contribute to the final extent of oedema, which in turn will contribute to errors in oedema quantification. As in Figure 3 and Figure 7, false positive pixels may be included, illustrating the importance of accurate LV boundary segmentation for automatic oedema quantification. If the manual approach to delineation of endo- and epicardial boundaries is used then the Dice similarity for oedema estimation is 0.8±0.05, higher than using automatically delineated LV boundaries, also close to the value in the inter-observer study.

Gudbjartsson et al. [31] suggested that for magnitude MR images, if signal to noise ratio, (SNR: defined as A/σ, A is the mean pixel intensity in the absence of noise, and σ denotes the standard deviation of the noise), is less than 1, the distribution of MR image intensity can be approximated by Rayleigh distribution; while when SNR as small as A/σ=3, it can be approximated by Gaussian distribution. Four random patients were selected for evaluating SNR. σ was averaged from the original MR images measured from regions in background air. After windowing process, σ was assumed to be the same, the averaged pixel intensity *M* in oedema regions was 103, and 3.85 in unaffected regions. Then SNR for oedema regions was $A/\sigma =\sqrt{\left|M^{2}-\sigma^{2}\right|}\;/\sigma =5.328$, larger than 3, for unaffected regions: *A/σ*=0.98. The analysis indicates Rayleigh-Gaussian distribution is applicable in this study. Furthermore, the comparison with Kadir’s method [17] shows that our approach performs better for oedema quantification even though the results from Kadir’s work produced acceptable Dice similarity scores. Johnstone et al [21] used a Gaussian mixture model to fit the myocardium intensity histogram with manual delineated LV boundaries and the agreement using Dice similarity was low (0.5). Since Kadir’s work and Johnstone’s work are based on the assumption that myocardium intensity has a Gaussian distribution, the higher agreement in our study also may suggest that the Rayleigh-Gaussian distribution is a good approximation for bright-blood T2-weighted oedema images. Our use of the Rayleigh-Gaussian distribution approximation on bright blood T2-weighted oedema images is novel and more studies are needed to further evaluate the performance and applicability of this approach.

By incorporating a prior model of the maximal extent of user defined culprit, Sjogren et al [22] improved the performance of Johnstone’s method for oedema segmentation from base to apex. However Sjogren’s method still required manual delineation of endo/epi-cardial boundaries and the oedema region was detected based on pre-defined regional analysis rather than the pixel-wise method used widely in other studies. Our method provides comparable results with similar bias of -0.4±4% with automatically detected LV boundaries, but lower Dice similarity coefficient 0.74±0.06. The lower Dice similarity coefficient in our study could be due to the pixel wise analysis for initial oedema segmentation rather than the regional analysis and the less accurate automatically delineated LV boundaries compared to manually segmented LV boundaries. Other factors may also be relevant such as variation in pathology between subjects (e.g. myocardial haemorrhage within the infarct zone), and image artefacts. In fact, some prior information is included in our method by retaining the option to alter the grey-scale window and level and observer judgement on the presence of oedema. Considering the superior diagnostic performance of bright blood T2-weighted oedema imaging over other methods (e.g. dark blood STIR MRI) [7], we still think observer input is important to avoid automated delineation of artefacts. Currently most computerized methods for oedema quantification do not take advantage of the information from image windowing [21,22], which we believe facilitates image assessment as illustrated in Figure 2(b).

After thresholding, alternate morphological filtering was applied to produce smoothed oedema regions without changing the overall shape. A kernel size of 2 pixels was used in the beginning for the closing and opening process followed by a kernel size of 5 pixels for the closing process. The kernel size of 5 pixels for the last closing process is in line with the approach by Hsu et al. (5 mm) [15]. The morphological filtering might still not be able to close out a signal void within the oedema region, as in Figure 3(d). Accordingly an algorithm for detecting dark zones, which potentially may be myocardial haemorrhage, is desired. In Johnstone’s study [21], a hyperintense region of possible interest but <1 g mass was considered to be noise and excluded from oedema quantification. In our dataset, since each oedema region is approximately perpendicular to the long axis of the LV and the slice interval is 10 mm, if mid-ventricular dimensions are adopted (cavity diameter: 45 mm, wall thickness: 10 mm), then the average oedema mass at each slice is approximately 6 g for manual delineation with an assumed density of 1.05 g cm^3^. In oedema region feature analysis in our study, approximately one-fifth of the main oedema area was considered to be a critical threshold for discrimination from noise. This threshold area corresponds to an average mass of 1.2 g. To improve the post-processing, minimum distance constraint criteria with the main oedema region was applied. By increasing the degree to 20 in the third step of feature analysis, and in the second step, the degree was changed to 40 at the same time. The result showed that the oedema extent was 27±8% with a mean Dsc of 0.73±0.06 related to the manual quantification. This analysis indicates that the oedema extent may not be sensitive to the choice of the minimum distance constraint. If no any feature analysis was applied, then the mean oedema extent was 30±8% (Dsc: 0.7±0.06), suggesting that oedema feature analysis is essential. Another potential source of error for overestimation of the oedema area is incorrect placement of the endocardial border within the bright LV cavity blood pool. In future a dark blood T2-prepared CMR method with steady state free precession readout might overcome this problem.

In this study, we intended to minimize user-inputs for oedema quantification, therefore there is no manual correction for automatic LV boundary segmentations and oedema delineations. As shown in Figure 3 and Figure 7, false positive and positive false pixels are present for reasons including (1) inaccurate automatic LV boundary segmentation, and (2) large dark areas inside the oedema regions. We believe that with necessary manual correction, the accuracy of oedema quantification will be highly improved.

In order to use our method attention is needed for: (1) the windowing process, which could introduce variability due to the observer’s experience. Our sensitivity study of the windowing process shows that the automatic approach is able to quantify oedema region consistently if the observer is trained properly on T2-weighted oedema images. (2) The definition of optimal threshold value, which might be different from 0.7 when actual oedematous regions are available for comparison, thus it needs to be carefully selected for different studies. (3) The oedema region feature analysis, which may not be applied to patients such as with myocarditis. Other limitations: (1) the manual input in our method involves user-defined adjustment of the greyscale level. Potentially, future technical developments may enable this step to be removed. Secondly there is no automatic decision making step for the presence of oedema. However, we suggest this aspect should be considered a strength of the method since physicians’ judgment is still retained; (2) Improvement in methods for automatic LV boundary segmentation and dark zones detection (such as haemorrhage) within the oedema regions are needed for more accurate oedema quantification; (3) 3D anatomical structure information of oedema, culprit coronary artery assignment [22] and myocardial infarction could be integrated in order to optimize the post-processing and segmentation results; (4) our method has been tested in a reasonably large cohort of patients with acute myocardial infarction. The performance of our method in other pathological conditions, such as acute myocarditis, needs further evaluation. Future studies should validate the method in animal experiments and in a larger cohort of acute MI patients.

## Conclusions

A highly automatic scheme has been developed for oedema quantification based on bright blood T2-weighted CMR. The method includes a level set model for automatic left ventricular wall segmentation and oedema delineation by using a Rayleigh-Gaussian statistical mixture model. The results show that the method could produce accurate delineation of oedema regions on bright blood T2-weighted CMR compared with manual oedema quantification. The method has some user-involvement and can be rapidly performed. We have also shown that reliable and accurate left ventricular wall boundary segmentation is essential.

## Additional file

## Abbreviations

CMR: Cardiac Magnetic Resonance; MI: Myocardial infarction; SDs: Standard deviations; LGE: Late Gadolinium Enhancement; pPCI: Primary percutaneous coronary intervention; STEMI: ST elevation myocardial infarction; SSFP: Steady state free precession; TSE-SSFP: Turbo spin echo steady state free precession; PSIR: Phase sensitive inversion recovery; MRI: Magnetic resonance imaging; PSIR-FLASH: PSIR turbo fast low-angle shot; Dsc: Dice similarity coefficient

## Competing interests

The authors declare that they have no competing interests.

## Authors’ contributions

HG performed the study design, algorithm development, result analysis and drafted the manuscript. KK participated in the study design, algorithm development (especially in left ventricular boundary segmentation) and data analysis. AP scanned the patients, contributed to the data analysis. JS participated in the study design and revised manuscript. CB participated in the study design, patient scan, and manual quantification and revised manuscript. All authors read and approved the final manuscript.

## Supplementary Material

Additional file 1 **Appendix 1.** The left ventricular (lv) endocardial and epicardial wall boundaries were segmented by using an automatic approach based on a variational level set method (vlsm), including lv centre definition for level set initialisation and boundary detections by vlsm. Click here for file
