# Supplementary material for: Highly automatic quantification of myocardial oedema in patients with acute myocardial infarction using bright blood T2-weighted CMR
Source: J Cardiovasc Magn Reson. 2013 Mar 30;15(1):28. doi: 10.1186/1532-429X-15-28 (PMC3621376; doi:10.1186/1532-429X-15-28)
Supplement: Additional file 1 — Appendix 1. The left ventricular (lv) endocardial and epicardial wall boundaries were segmented by using an automatic approach based on a variational level set method (vlsm), including lv centre definition for level set initialisation and boundary detections by vlsm. [file 1532-429X-15-28-S1.doc]

Appendix 1

The left ventricular (LV) endocardial and epicardial wall boundaries were segmented by using an automatic approach based on a variational level set method (VLSM), including LV centre definition for level set initialisation and boundary detections by VLSM.

## Automatic initialization of level set

The automatic left ventricular centre point detection presented in [28] was performed to detect the centre of the left ventricular cavity in short axial cardiac MRI images by employing the features that: a) the left ventricle is located a little to the right from the centre of the image plane and b) the blood cavity of the left ventricle appears as a bright area surrounded by dark myocardial wall as in Fig 2(a). Fuzzy membership functions were used to represent the spatial information and intensity information of the left ventricle. By applying fuzzy operators for intersections to select the higher membership degree, the intersection point was defined as the left ventricular cavity centre and used to initialize the first level set boundary by using conventional region growing method.

## Automatic LV wall segmentation

A variation level set method (VLSM) without-re-initialization [30] was used for left ventricular wall segmentation. The evolution equation of the traditional level set formulation can be written in a general form,

(a.1)

where *F* is the speed function, which depends on the imaging data *I* and level set function. The moving front *C* can be represented by the zero level set as

(a.2)

*C* can represent the endocardial and epicardial boundaries of the left ventricular wall.

For the traditional LSM, is required to be kept close to a signed distance function during the evolution, therefore re-initialization is required constantly. However the re-initialization procedure can be very complicated and time consuming and has significant side effects. In order to overcome those difficulties, the evolution Eq. a.1 is redefined as

(a.3)

where $\omega$ is

(a.4)

The first term in the right hand side of Eq. a.4 is the measurement of the distance of to a signed distance function, which will eliminate the re-initialization of during level set evolution, and $\alpha$>0. The second and third terms in the right hand side of Eq. a.4 are the energy terms which will drive the motion of the zero level curve of to the desired boundaries. $\beta>0$ and $\gamma$ is a constant. $\delta$ is the univariate Dirac function and *H* is the Heaviside function. $g$is the edge indicator, defined as

$g=\frac{1}{1+\left|(\nabla G_{\sigma})*I\right|^{2}}$ (a.5)

$G_{\sigma}$is the Gaussian kernel with standard deviation $\sigma$and * is the convolution operator.

The segmentation procedure was carried out on a slice-by-slice basis as follows:

1. Pre-processing MRI edema images, using the combination of geodesic morphological operation and a conventional anisotropic diffusion process to reduce the isolated papillary muscles and intensity heterogeneity;
2. LV cavity centre detection by using Fuzzy membership analysis;
3. Endocardial boundary tracking by VLSM, followed by the convex hull algorithm to exclude papillary muscles attached to the LV wall;
4. Initialization of VLSM for epicardial boundary segmentation by expanding the endocardial boundary outward slightly. The averaged LV wall thickness in the septum region is assessed in order to constrain the epicardial boundary tracking, which helps to stop boundary leaking;
5. Epicardial boundary tracking by VLSM.
